# Sensing Technologies for Outdoor/Indoor Farming

**DOI:** 10.3390/bios14120629

**Published:** 2024-12-19

**Authors:** Luwei Wang, Mengyao Xiao, Xinge Guo, Yanqin Yang, Zixuan Zhang, Chengkuo Lee

**Affiliations:** 1 Department of Electrical and Computer Engineering, National University of Singapore, Singapore 117576, Singapore; 2 Center for Intelligent Sensors and MEMS (CISM), National University of Singapore, Singapore 117583, Singapore; 3 Research Center for Sustainable Urban Farming (SUrF), National University of Singapore, Singapore 117558, Singapore; 4 National University of Singapore Suzhou Research Institute (NUSRI), Suzhou Industrial Park, Suzhou 215123, China; 5 NUS Graduate School–Integrative Sciences and Engineering Programme (ISEP), National University of Singapore, Singapore 119077, Singapore

**Keywords:** plant health monitoring, wearable sensors, flexible sensors, multimodal sensors, outdoor/indoor farming

## Abstract

To face the increasing requirement for grains as the global population continues to grow, improving both crop yield and quality has become essential. Plant health directly impacts crop quality and yield, making the development of plant health-monitoring technologies essential. Variable sensing technologies for outdoor/indoor farming based on different working principles have emerged as important tools for monitoring plants and their microclimates. These technologies can detect factors such as plant water content, volatile organic compounds (VOCs), and hormones released by plants, as well as environmental conditions like humidity, temperature, wind speed, and light intensity. To achieve comprehensive plant health monitoring for multidimensional assessment, multimodal sensors have been developed. Non-invasive monitoring approaches are also gaining attention, leveraging biocompatible and flexible sensors for plant monitoring without interference with its natural growth. Furthermore, wireless data transmission is crucial for real-time monitoring and efficient farm management. Reliable power supplies for these systems are vital to ensure continuous operation. By combining wearable sensors with intelligent data analysis and remote monitoring, modern agriculture can achieve refined management, resource optimization, and sustainable production, offering innovative solutions to global food security and environmental challenges.

## 1. Introduction

As the global population continues to increase, the demand for grain has surged significantly. Meeting this increasing need calls for improvements in both crop yield and quality [1,2,3]. Plant health plays an important role in determining crop quality and yield; however, many factors influence plant health, including environmental stress and plant diseases. High temperatures, drought, excessive rainfall, and frost [4,5,6,7] can lead to issues like heat stress, water deficiency, and root damage which all negatively impact plant growth and productivity. By monitoring microclimates such as light intensity, temperature, and humidity, plant growth conditions can be optimized to create a more suitable environment, ultimately boosting crop yields [8,9,10]. For example, the optimum temperature of lycopene formation of tomato is 24 °C and when provided with a higher temperature, the lycopene is not formed [11]. In addition to environmental challenges, plant diseases [12] like late blight disease [13], wheat streak mosaic [14], and powdery mildew [15] pose significant threats by causing stunted growth, leaf yellowing, and even death. Plants affected by diseases or damage release specific metabolites, VOCs, and undergo a change in water content in a manner significantly different from healthy plants [16]. By monitoring these characteristics, early diagnosis of plant disease can be achieved, leading to effective prevention and control [17,18]. Other physiological indicators, such as water content and elongation, are also critical markers of plant health, providing valuable insights into the overall growth status and vitality of plants.

Based on the sensing mechanisms, different sensing technologies for plant health monitoring can be classified into piezoelectric ultrasonic sensors, optical-based sensors, strain sensors, impedimetric sensors, and chemical sensors. Piezoelectric ultrasonic sensors enable precise monitoring of plant health by detecting changes in water stress, elasticity, and structural properties, offering valuable insights into plants’ physiology and water status [19,20,21,22,23,24,25]. Optical-based sensors provide real-time assessment of plant health by analyzing spectral reflectance, fluorescence, and imaging data to detect nutrient status, stress conditions, and disease [26,27]. These sensing technologies have a good detection effect on the growth dynamics of plants, but some of these sensors are relatively rigid and usually difficult to adapt to fragile and soft plant structures, which may affect the normal growth of plants in long-term monitoring [28]. With the development of sensing technologies and device fabrication, more and more non-invasive wearable plant sensors are designed [29,30,31]. Due to their stretchability and flexibility, these sensors can be attached to the surface of plant leaves or stems without interference with plant growth. The materials used for these sensors are crucial, as they must be non-toxic and biocompatible to ensure plant health growth. Ideally, these materials should also allow light transmission to support the photosynthetic process and maintain environmental cues necessary for stomatal function, enabling the stomata to open and close as needed [32,33]. This ensures that key processes like photosynthesis and respiration remain undisturbed, thereby promoting healthy plant growth. To provide a comprehensive insight into plant health and environmental interactions, various wearable sensors are available to monitor different plant parameters, including physical, chemical, and biological signals, enabling precise, real-time monitoring. Wearable strain sensors can track the growth rate and morphological changes of plants, while impedance sensors evaluate the moisture status of plants by monitoring impedance changes [34,35,36]. At the same time, chemical sensors can detect the VOCs [37] released by plants; for example, they can identify ethylene signals [38] when fruits ripen or specific compounds are released by plants with diseases. All of the parameters are important for plant growth; multimodal sensor systems can not only monitor the growth status of plants, but also track changes in temperature, humidity, light intensity, and harmful gases in the environment, comprehensively improving the accuracy and breadth of plant health monitoring [32,39,40]. Wearable sensors with wireless transmission capability are particularly important in modern agriculture, these sensing systems can transmit data to the host wirelessly, allowing real-time monitoring and human intervention. For example, alarms can be triggered based on data regarding the size and maturity of fruits collected by wireless sensor systems. This helps prompt farmers to harvest fruits at the optimal time, thereby ensuring higher crop quality and yield. Energy supply [41] is another issue that needs to be considered in farm plant monitoring especially for outdoor agriculture. Traditional energy supply can be costly, not reliable in extreme environments, and often involves materials that are harmful to the environment. Self-sustainable plant Internet of Things (IoT) monitoring systems provide a feasible approach that not only helps solve the energy-supply problem but also ensures the stability and durability of sensors in extreme environments, thereby further improving the monitoring efficiency of outdoor agriculture.

**Figure 1:**
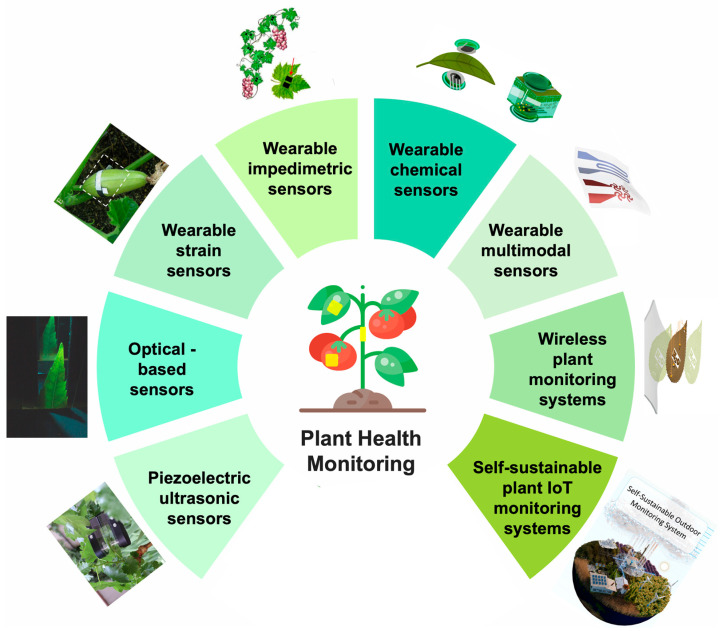
Overview of wearable sensing technologies for plant health monitoring: variable sensing technologies including piezoelectric ultrasonic sensors [20], optical-based sensors [44], wearable strain sensors [35], wearable impedimetric sensors [17], and wearable chemical sensors [45] play a crucial role in plant health monitoring. Advanced plant-monitoring systems that integrate multimodal sensors [32], wireless data transmission [46], and self-sustainability [47] are increasingly popular.

This article introduces sensing technologies based on different working principles, which are used to monitor plant physiological indicators, plant microclimate, plant signaling molecules, etc., to ensure the healthy growth of plants (Figure 1). With the rapid development of plant-sensing technologies, the agricultural field has entered a new era of more refined management and efficient production by developing wireless transmission and self-powered IoT systems. In addition, combined with intelligent data analysis and remote monitoring technology, agricultural production can achieve more efficient resource management and accurate decision-making, promote the development of sustainable agriculture, and provide new solutions to global food security and environmental challenges [42,43].

## 2. Piezoelectric Ultrasonic Sensors for Plant Monitoring

Piezoelectric ultrasonic sensors for plant monitoring play a significant role in understanding plant health and physiology through non-destructive, precise ultrasonic techniques. These sensors are employed for various applications, including detecting water stress in plants by measuring ultrasonic velocity changes in response to dehydration through the alteration of acoustic impedance caused by changes in water content and tissue elasticity [19,20,23,48,49,50,51,52]; assessing plants’ elasticity through ultrasonic resonance, which helps determine the mechanical properties of leaves by measuring the frequency shifts and damping factors of resonant vibrations under controlled excitation conditions [22,53,54]; characterizing xylem and other structural properties using ultrasonic waves to understand water-transport mechanisms via analysis of acoustic emissions generated by cavitation events and structural vibrations [48,55,56,57]; and utilizing broadband ultrasonic spectroscopy for multi-parameter analysis, allowing comprehensive insights into plant health, including both structural and physiological conditions by integrating spectral analysis with machine learning for enhanced parameter extraction [21,58,59,60].

**Figure 2:**
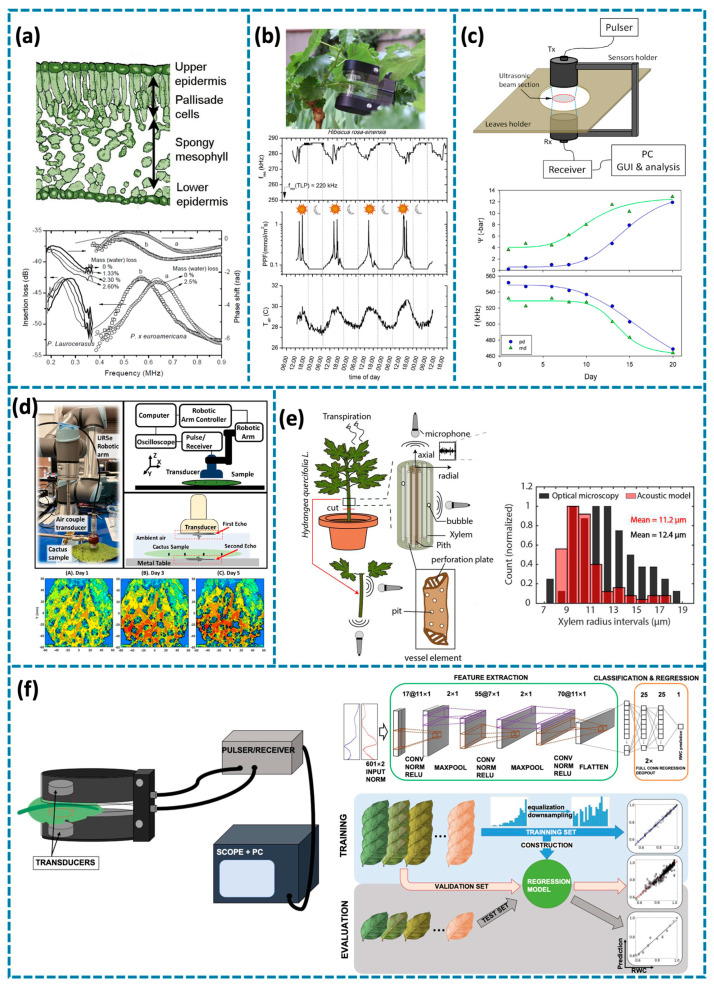
Piezoelectric ultrasonic techniques for plant monitoring. (**a**) Ultrasonic propagation in leaves, showing anatomy, wave behavior, and water content effects [19]. (**b**) Environmental monitoring via ultrasonic pulse and frequency changes [20]. (**c**) Real-time crop water needs assessment during irrigation [23]. (**d**) Elasticity measurement with a robotic ultrasonic transducer [53]. (**e**) Xylem monitoring with ultrasonic pulses, stem structure, and vessel size distribution [48]. (**f**) Leaf water content prediction using deep learning and ultrasonic analysis [59].

Water stress is one of the primary factors that can significantly affect plant growth and productivity. Accurate detection and monitoring of water stress are essential for maintaining plant health, particularly in agricultural settings. Ultrasonic techniques have emerged as effective tools for non-invasive monitoring of water stress in plants, providing valuable insights into plant water status and helping to optimize irrigation practices [48,52]. These techniques leverage the relationship between water content and ultrasonic wave propagation characteristics, such as velocity and attenuation, to detect subtle changes in plant hydration levels. Air-coupled ultrasound is particularly advantageous for assessing water status in leaves, offering a real-time, non-destructive means to monitor plant water content. Air-coupled ultrasound was shown to effectively assess leaf water status, enabling continuous, non-invasive plant monitoring [19]. In these applications, the ultrasonic transducer generates airborne ultrasonic waves that pass through the leaf tissue, and the received signals are analyzed to determine water content based on changes in acoustic properties. As shown in Figure 2a, T. Gómez Álvarez-Arenas et al. provided a visual summary of key findings from the study, including the internal structure of the leaf, such as epidermis and mesophyll layers, and their respective impacts on ultrasonic wave propagation. The figure illustrates the principles of wave reflection, refraction, and transmission through different tissue layers, highlighting how water loss alters these dynamics. Further advancements in ultrasonic sensing involved using ultrasonic waves to monitor plant responses to environmental changes [20]. By capturing variations in plant behavior due to changing water availability, ultrasonic sensing proved valuable for early stress detection. This non-invasive approach ensures that plants can be monitored continuously, which is crucial for precision agriculture. As shown in Figure 2b, M. D. Fariñas et al. illustrated different aspects of this study. These images help to visualize how ultrasonic sensing can effectively capture changes in plant physiology due to environmental factors, aiding in the early detection of water stress and enabling better management of plant health. The figure demonstrates the correlation between ultrasonic parameters, such as Q-factor and resonant frequency, and physiological changes induced by water stress, providing a clearer visualization of plant responses under varying hydration conditions. In 2016, ultrasonic sensing was applied by T. Gómez Álvarez-Arenas et al. specifically to determine crop water needs, thereby enhancing precision irrigation [23]. The method relied on precise measurement of resonant frequency shifts in leaves to estimate their relative water content (RWC) and turgor pressure. The study proposed a feedback system for irrigation scheduling based on real-time ultrasonic measurements, optimizing water usage. The study used ultrasonic sensors to identify the optimal timing and amount of irrigation, leading to more efficient water use, particularly in areas with limited water resources, as shown in Figure 2c. Recent studies have further expanded on these applications. For instance, in 2021, ultrasonic parameters were shown to be rapid and reliable indicators of water stress in citrus plants [50]. Contact-less, high-frequency ultrasonic techniques, as developed by Fariñas et al. in 2022, also demonstrated their capability in detecting plant water content non-invasively [49]. Additionally, Oletic and Bilas introduced an energy-efficient piezoelectric sensor front-end designed to acquire ultrasonic emissions associated with water stress in plants [51]. The study focused on monitoring acoustic emissions from xylem embolisms, which occur due to cavitation in water transport vessels under water stress. This technique provided a field-deployable solution for capturing real-time water stress indicators. The integration of such piezoelectric sensor systems into plant monitoring allows for real-time, energy-efficient detection of water stress, improving the potential for precision agriculture in challenging environments. 

Plants’ elasticity is a crucial mechanical property that reflects how plant tissues deform under stress and return to their original shape. It is closely linked to water content, as dehydration causes tissues to stiffen and their elastic modulus to increase [22,53,54]. Ultrasonic resonance techniques enable non-invasive assessment of elasticity by exciting mechanical vibrations in plant tissues and analyzing the resonant frequency shifts and damping characteristics. To assess plants’ elasticity non-invasively, ultrasonic resonance techniques have been developed, providing valuable insights into plant health and physiological changes. In 2012, air-coupled ultrasonic resonant spectroscopy was introduced to evaluate leaf elasticity, revealing a strong relationship between water content and stiffness [54]. The methodology involved measuring resonant frequency variations as an indicator of mechanical changes in tissues caused by hydration levels. This method proved highly sensitive to changes in tissue stiffness, making it an effective tool for monitoring plant health under varying water conditions. Ultrasonic waves were used to quantitatively measure the elastic modulus, offering a clearer understanding of how plant tissues respond to dehydration. Building on this, ultrasonic resonance was applied to monitor diurnal changes in leaf elasticity, particularly in grapevines [22]. These changes were linked to osmotic adjustments driven by photosynthesis, demonstrating that ultrasonic techniques could capture subtle physiological variations throughout the day. This capability proved valuable for understanding plant responses to environmental fluctuations and managing crop health. In 2022, Yang et al. advanced the field by integrating robotic systems with air-coupled ultrasonic transducers for precise, non-contact measurements of tissue elasticity [53]. This study further enhanced measurement accuracy by developing a non-contact ultrasound system using a novel air-coupled transducer, enabling effective monitoring of plant tissue elasticity without direct contact. This approach was especially suitable for delicate plant tissues, as shown in Figure 2d. This modern, non-contact approach improved measurement precision and allowed for detailed mapping of plant tissue properties, offering a comprehensive view of plant health over time.

Xylem is crucial for water transport within plants, and understanding its structure is essential for assessing plant health, particularly under water stress conditions [48,55,56,57]. Ultrasonic techniques have proven highly effective in non-invasively characterizing xylem structures, offering valuable insights into plant water-transport mechanisms. The application of ultrasound pulse emission spectroscopy enables detailed analysis of xylem vessel dimensions and elasticity by interpreting the acoustic signatures generated during cavitation events. Ultrasound pulse emission spectroscopy, developed in 2021, demonstrated its ability to evaluate xylem vessel dimensions and elasticity [48]. As shown in Figure 2e, S. Dutta et al. provided a visual overview of this study. The figure highlights how resonant vibrations correlate with structural properties of xylem vessels, offering insights into their efficiency in water transport. By analyzing resonant vibrations, researchers gained insight into the efficiency of water transport in plants. This non-destructive approach is particularly useful for evaluating plant water management under different conditions, providing a window into the internal dynamics of plant physiology. Acoustic emissions, as demonstrated in 2016, have also been used to detect drought-induced cavitation events in xylem [57]. These emissions arise from the rapid collapse of water columns under high tension, serving as direct indicators of drought stress. Ultrasonic techniques enable real-time detection of these cavitation events, helping researchers understand plant responses to water scarcity and identify vulnerable species. Further advancements were made in 2020 with the development of micro-mechanical cantilever resonators to detect cavitation emissions, enhancing sensitivity in identifying water stress-related events and contributing to improved drought management strategies [55]. Ultrasonic sensors, validated in 2021, demonstrated their sensitivity in detecting xylem acoustic emissions, confirming their effectiveness for real-time monitoring of drought-induced physiological changes [56].

Broadband ultrasonic spectroscopy is a powerful tool for multi-parameter analysis of plant health, allowing the simultaneous assessment of various physiological attributes [21,58,59,60]. The technique’s versatility lies in its ability to capture a wide frequency range of ultrasonic signals, which provides detailed information on plant structure and water content. In 2010, researchers utilized air-coupled broadband ultrasonic spectroscopy to non-invasively determine leaf water status, enabling continuous monitoring of plant health across diverse environmental conditions [58]. By capturing broadband ultrasonic signals, they were able to extract detailed information about water content and the structural integrity of plant tissues. These measurements were particularly beneficial in identifying early signs of stress, such as reduced water retention or changes in cell wall elasticity. The approach was further expanded in 2013, exploring the reflectivity of ultrasonic waves in grapevine tissues [60]. This analysis provided insights into the plant’s internal water relations, enabling the monitoring of water movement and retention within plant tissues—key factors in understanding how plants respond to water availability. Meanwhile, ultrasonic sensing for plant water needs, developed in 2015, offered valuable insights into how ultrasonic methods can enhance precision irrigation [21]. Applying broadband ultrasonic spectroscopy to entire ecosystems enables a deeper understanding of plant-environment interactions and the assessment of vegetation health across different types. In 2019, broadband ultrasonic spectroscopy was combined with deep learning to introduce a novel method for estimating the relative water content in plant leaves [59]. This integration of machine learning algorithms allowed for more accurate interpretation of complex ultrasonic spectra, improving the reliability of water content predictions. By applying deep learning algorithms to resonant ultrasonic spectra, M. D. Fariñas et al. significantly improved the accuracy and reliability of water content estimation, as shown in Figure 2f. This integration of advanced sensing technology and data-driven analysis enhances precision in assessing plant hydration, contributing to more effective agricultural management.

## 3. Optical-Based Sensors for Plant Analysis

Optical-based sensors for plant analysis offer crucial tools for understanding and managing plant health by providing non-invasive, precise, and real-time monitoring [61,62,63,64]. These sensors are utilized for various applications, including assessing plants’ nitrogen status through spectral reflectance analysis, which helps determine nitrogen sufficiency by measuring chlorophyll content [44,64,65,66,67]; evaluating plant health via fluorescence emission, which provides insights into nutrient deficiencies and stress conditions [63,68,69,70,71,72,73,74,75,76,77]; monitoring greenhouse gases like carbon dioxide (CO_2_) to optimize photosynthesis and environmental factors for improved growth [70,78,79,80]; and detecting plant stress and disease using technologies like multispectral, hyperspectral, and thermal imaging to enable early intervention before visible symptoms emerge [61,62,81,82,83,84,85,86,87,88]. Furthermore, integrating machine learning with optical sensor data enhances the accuracy and efficiency of plant health assessments, providing data-driven insights for precision agriculture [89].

**Figure 3:**
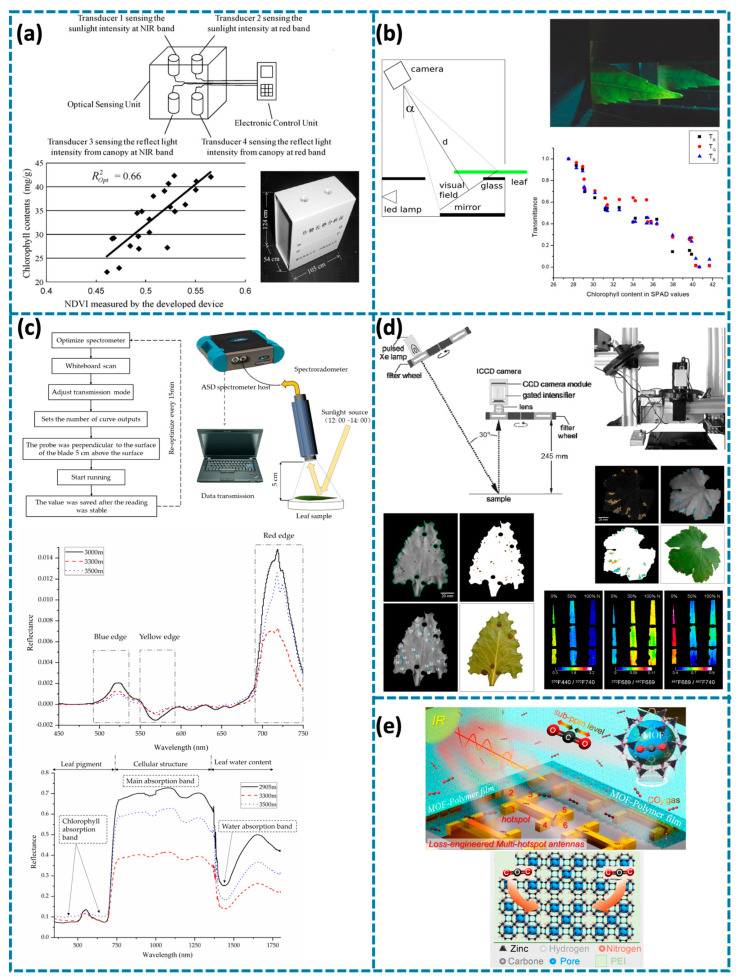
Optical sensors for plant monitoring. (**a**) Hand-held NDVI sensor with components and chlorophyll correlation [67]. (**b**) Reflectance and transmittance method for chlorophyll estimation, including setup and leaf sample analysis [44]. (**c**) Spectral reflectance of Quercus aquifolioides at various altitudes, highlighting key absorption bands [66]. (**d**) Multi-color fluorescence imaging system for plant stress detection with setup and fluorescence images under different excitations [76]. (**e**) MOF-polymer system for CO_2_ detection, featuring infrared (IR) absorption enhancement and selective adsorption [80].

Assessing plant nitrogen status through spectral reflectance analysis involves using different wavelengths of light to analyze plant tissues, with chlorophyll content often acting as an indicator of nitrogen levels [44,65,66,67]. A hand-held optical sensor was developed by D. Cui et al. to measure the normalized difference vegetation index (NDVI), effectively correlating chlorophyll content with nitrogen levels in a field setting, as shown in Figure 3a [67]. Similarly, an evaluation in 2014 employed optical sensors to assess canopy reflectance and leaf chlorophyll content, revealing a strong correlation between NDVI and nitrogen sufficiency in crops like muskmelon [65]. In 2008, the concept of using signature spectral cues, such as changes in leaf reflectance spectra, was introduced to non-invasively monitor plant stress and nutrient levels, including nitrogen [64]. It emphasized the relationship between specific spectral signatures and plant health, and the potential application of these technologies under field conditions. In 2018, another method was introduced using reflectance and transmittance data from plant leaves to estimate chlorophyll content, highlighting a non-destructive approach to evaluating nitrogen levels [44]. M. Pérez-Patricio et al. utilized an optical setup designed to capture both reflectance and transmittance properties of leaves, providing a quick and accurate means to assess chlorophyll, which is directly related to nitrogen status, as shown in Figure 3b. The data points clearly show an inverse relationship between transmittance and chlorophyll content, which serves as an indicator of nitrogen levels in the plant. This non-destructive method allows for continuous monitoring of plant health, supporting effective nitrogen management in crops.

Spectral reflectance characteristics of Quercus aquifolioides at different altitudes, analyzed in 2020, demonstrated how environmental factors influence chlorophyll content and, consequently, the nitrogen status of the plants [66]. J. Zhu et al. used an analytical spectral device (ASD) spectrometer to measure reflectance from different leaf samples, providing detailed spectral data across a range of wavelengths, as shown in Figure 3c. These characteristics are critical in understanding how altitude influences chlorophyll content, as seen from the spectral responses, with noticeable variations in the red and near-infrared regions depending on altitude. The results of this study indicated that the reflectance spectra varied significantly with altitude, especially in the visible and near-infrared bands, which are directly linked to chlorophyll content and nitrogen status.

Fluorescence emissions from plant tissues can be used to assess their physiological condition, particularly regarding nitrogen status [68,71,72,73]. Chlorophyll fluorescence is a key indicator in this process, providing insights into how effectively a plant is utilizing nitrogen [69,70,74]. The Multiplex sensor, introduced by Lejealle et al. in 2010, demonstrated its application in assessing nitrogen content in turfgrass [77]. The sensor’s ability to record chlorophyll fluorescence and related fluorescence indices enabled it to assess the nitrogen balance index (NBI), which is a critical indicator of nitrogen sufficiency. This study showed the feasibility of using fluorescence-based measurements for site-specific nitrogen management in turfgrass, leading to improved plant health and optimal fertilization practices. Additionally, in 2012, the Multiplex hand-held fluorescence sensor was introduced to measure chlorophyll fluorescence under different excitation lights, including ultraviolet (UV), blue, green, and red [75]. This allowed for the monitoring of nitrogen status in corn by using various fluorescence ratios, which proved effective in assessing chlorophyll and nitrogen content in the plants at different growth stages. In 2014, an advanced multi-color fluorescence imaging system was designed to detect both biotic and abiotic stresses in leaves [76]. S. Konanz et al. used different excitation wavelengths to create fluorescence images, which allowed researchers to analyze plants’ stress symptoms across entire leaf areas. The capability of multi-color fluorescence imaging to differentiate between stress types was particularly valuable for understanding how nitrogen deficiency impacts overall plant health, as shown in Figure 3d. The ability to map stress responses visually across the entire leaf surface offered a significant advantage in monitoring nitrogen-related stress, as well as other forms of biotic and abiotic stress. This fluorescence imaging system provided a non-invasive method to monitor plant health more accurately and holistically. In 2018, proximal optical sensors, including fluorescence-based sensors like the Dualex and Multiplex, were evaluated for their use in nitrogen management in vegetable crops [63]. The sensors measure chlorophyll fluorescence and other related parameters to provide insights into the nitrogen status of crops in real time. This fluorescence-based approach allows for precise nitrogen fertilization, optimizing the nutrient application to maintain plant health. These studies illustrate the importance of fluorescence emission in evaluating plant health, particularly in detecting nitrogen deficiencies, monitoring overall plant physiological status, and enabling sensitive detection of nitrogen-related changes in plant tissues. Fluorescence techniques, including advanced imaging systems and sensors, provide detailed, non-destructive insights.

Monitoring greenhouse gases is a critical aspect of ensuring optimal plant growth conditions, particularly in controlled environments like greenhouses. Monitoring CO_2_ levels is essential for maintaining the balance between CO_2_ assimilation and photosynthetic efficiency, especially in plants with variable nitrogen availability [70]. Nitrogen deficiency in maize plants was shown to impact photosynthetic CO_2_ assimilation, chlorophyll fluorescence, and susceptibility to photoinhibition. The deficiency reduced the plants’ capacity for CO_2_ assimilation, increasing the risk of photodamage under high light conditions. These findings highlighted the importance of monitoring CO_2_ levels in conjunction with nutrient availability to prevent photoinhibition and maintain photosynthetic efficiency. In 2022, the integration of metal-organic frameworks (MOFs) with optical sensors was explored for detecting CO_2_, a crucial factor in plant growth within greenhouse environments [80]. H. Zhou et al. employed surface-enhanced infrared absorption (SEIRA) to achieve highly sensitive detection of CO_2_ concentrations, with a detection limit as low as 1 ppm. The use of MOFs allowed for selective adsorption of CO_2_, providing both high specificity and adsorption efficiency. This approach helped in adjusting greenhouse ventilation, ensuring that the CO_2_ levels were optimal for photosynthesis. By maintaining proper CO_2_ concentrations, the study demonstrated significant potential for enhancing crop yields in a controlled environment. The compact sensor design, capable of providing real-time data, made it well-suited for greenhouse applications, supporting efficient growth and effective gas management, as shown in Figure 3e. Optical-based sensors utilizing near-infrared (NIR) reflectance, developed in 2020, were used to monitor photosynthetic CO_2_ assimilation in plants, offering insights into photosynthetic efficiency under varying CO_2_ concentrations [79]. An overcoupled resonator design, introduced in 2024, was developed to enhance light-matter interactions, thereby improving CO_2_ detection capabilities [78]. This advanced resonator allowed for precise monitoring of CO_2_ levels, contributing to better greenhouse gas management to maintain optimal photosynthesis conditions in agricultural systems. These studies underscore the importance of CO_2_ monitoring for managing plant health and optimizing photosynthetic efficiency in greenhouses. The integration of MOFs and SEIRA technology was developed to provide sensitive, selective, and real-time CO_2_ monitoring, offering significant advantages in controlled agricultural settings. Proper CO_2_ detection not only supports efficient photosynthesis but also helps prevent issues like photoinhibition, especially under conditions of nutrient stress.

Detection of plant stress and disease is crucial for ensuring optimal crop health and productivity, particularly in the face of environmental stressors and pathogens [82,86,87]. Optical-based sensing technologies have emerged as effective tools for early detection, allowing for timely interventions in agricultural practices [61,62,81,85]. Hyperspectral reflectance combined with support vector machines (SVMs) was used to classify diseased and healthy sugar beet leaves [82]. The study demonstrated the effectiveness of hyperspectral reflectance in detecting disease at an early stage, before visible symptoms manifested. This capability is particularly valuable for managing diseases in precision agriculture, where early intervention is key to minimizing crop loss. In 2009, a comparison of commercial optical sensors was conducted in the context of precision viticulture [83]. The study highlighted the use of normalized difference vegetation index (NDVI)-based sensors to monitor crop health and detect specific diseases like phylloxera in vineyards. NDVI sensors provide valuable information regarding the vegetation status, allowing for the identification of stressed or diseased areas, which is essential for targeted management in high-value crops like grapes. In 2011, optical sensors were explored for estimating pasture quality, utilizing multispectral and hyperspectral remote sensing tools [88]. These sensors can assess vegetation characteristics, such as chlorophyll content, which are indicators of plant health and potential stress. This approach is beneficial for detecting early stress symptoms, allowing farmers to manage pasture health more effectively. R. R. Pullanagari et al. provided a visual representation of how optical sensors assess vegetation by measuring reflectance at different wavelengths, capturing important details about leaf structure and health. Non-destructive phenotyping of lettuce plants during early development stages was the focus of a study conducted in 2016 [61]. Optical sensors, including hyperspectral and chlorophyll fluorescence imaging, were used to identify phenotypic changes due to temperature and salinity stress. The ability to detect subtle changes in plant physiology at an early stage offers significant potential for improving stress tolerance through precise monitoring and management. Thermography was applied to detect drought response in maize [84]. By measuring leaf temperature, the researchers were able to confirm genotypic variation in response to drought stress. Thermal imaging is an effective tool for detecting water stress because leaf temperature changes are closely related to transpiration rates. This method provides a rapid, non-invasive way to monitor plant responses to drought conditions and identify drought-tolerant genotypes for breeding purposes.

Collectively, these studies demonstrate the effectiveness of optical-based sensing technologies in detecting plant stress and disease. Hyperspectral imaging, NDVI sensors, thermography, and fluorescence imaging have all shown promise in providing early indicators of plant health issues. By leveraging these tools, farmers and researchers can implement timely interventions, ultimately leading to improved crop productivity and resilience to stressors.

## 4. Wearable Strain Sensors for Plant Monitoring

**Figure 4:**
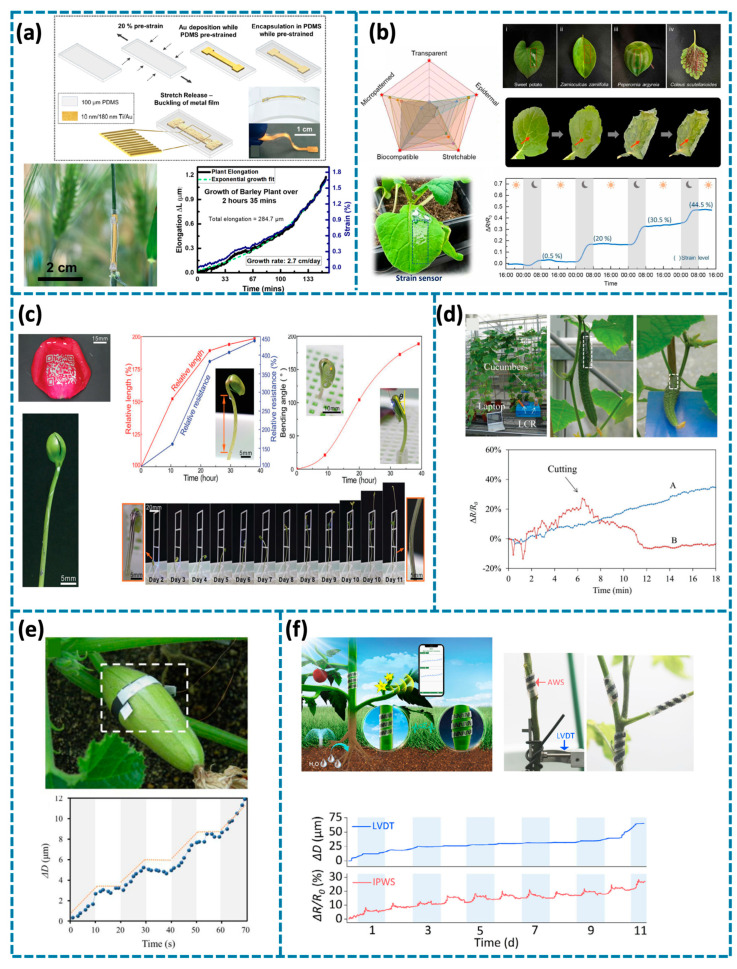
Strain sensors for plant monitoring. (**a**) Adhesive tape-assisted strain sensor for stem growth monitoring [94]. (**b**) Transparent and epidermal strain sensor for leaf growth monitoring [32]. (**c**) Substrate-less epidermal strain sensor for bean sprout seedling growth monitoring [95]. (**d**) Substrate-less epidermal strain sensor for fruit growth monitoring [96]. (**e**) Strain sensor wrapped on fruit for expansion monitoring [35]. (**f**) Tendril structure enabled self-adaptive wrapped strain sensor for wireless monitoring of plants’ pulse and growth [97].

Speaking of the health status of plants, the growth condition is one of the key representative parameters that can reflect the health status of plants [90]. Healthy plants exhibit normal growth patterns while plants stop growing under abiotic or biotic stress [91,92]. Currently, strain sensors show significant potential in real-time growth monitoring of the plants. The growth of plants imposes corresponding deformation on the strain sensors so that the resistance/capacitance will respond accordingly. Moreover, the strain sensors are required to be closely attached to the plants and deform with them without hindering their normal growth or metabolic activities. To enable the detection of plant growth through strain sensors, researchers utilize tapes to help fix the strain sensors on the plants [93]. As shown in Figure 4a, Joana et al. developed a stretchable strain sensor using polydimethylsiloxane (PDMS) as the substrate and bucked metal film as the sensing layer [94]. This strain sensor was attached to the germ of barley plants with two tapes fixing both terminals. It was recorded that a total leaf elongation of 284.7 μm was achieved for a total time of 2 h 35 min. Although the assistance of tapes allows the accurate monitoring of plant growth through the elongation of strain sensors, their relatively bulky and heavy structures may still limit the growth of plants and removal of such tapes may destroy the plants. Nowadays, epidermal strain sensors stand out owing to their ultrathin thickness, ultra-softness, and seamless attachment with the sensing targets as well as their non-invasive sensing capability. Therefore, epidermal sensors are widely utilized in plant growth monitoring, especially the fragile components, such as the leaves. For example, as shown in Figure 4b, Yang et al. developed an epidermal sensor with excellent light transparency based on poly(3,4-ethylenedioxythiophene): polystyrene sulfonate and PDMS as the sensing and substrate materials correspondingly [32]. This epidermal sensor can be seamlessly attached to various plant leaves without the assistance of any tapes. Seamless attachment with the plants was also secured even when the leaves were under different wilting stages. For practical application, the epidermal sensor was applied to the juvenile leaf of B. rapa to monitor its growth. Recorded relative resistance change of the strain sensor increased during a 3-day period, where the increase of the relative resistance change reveals the extension of the monitored leaves, i.e., growth. Leaf growth exhibited noncontinuous patterns, aligning with known growth behaviors where plants use stored energy to grow and elongate during nighttime while absorbing light for photosynthesis during the day.

In addition, strain sensors can also be developed directly on the plants for monitoring without substrates. This allows closer adhesion between plants and the sensors despite surface roughness, hairy or spiny, ensuring a more sophisticated and accurate recording of plant growth status. As shown in Figure 4c, Jiang et al. employed a hydroprinting process to develop substrate-less epidermal liquid-alloy-based electronics on different substrates, such as rose, living bean sprout seedlings, ballon [95]. The liquid alloys were patterned into different complex patterns to meet the requirements of different sensing purposes. For plant physiological monitor, the liquid alloy was printed on the living bean sprout seedling into a liner shape. Benefiting from the stretchability of the liquid alloy and the corresponding resistance difference under varying lengths, the printed sensor was used for bean growth monitoring. Consequently, the growing lengths of the bean sprout were calculated from the resistance change of the epidermal strain sensor. Meanwhile, the bending angle of the bean sprout was also monitored through the resistance change of the strain sensor. Besides the liquid alloy, Tang et al. proposed another substrate-less epidermal strain sensor by direct writing chitosan-based water ink on the leaves and fruits (Figure 4d) [96]. The strain sensor was successfully written on the surface of the cucumber despite its bumpy, hairy, and spiny surface. The increasing resistances of the written sensor suggested the increasing dimensions of both cucumber fruits. However, the curve of the cut fruit stopped increasing and dropped rapidly (curve B). In comparison, the curve of the other fruit continued growing (curve A). The dimension of the cucumber fruit decreased after cutting off from the stem, while that of the other fruit was not affected. It can be inferred that the fruit reduced its metabolism to deal with the lack of supplements, resulting in decreased dimension.

Besides elongation of stems, leaves, and fruits, the expansion of plants’ stems and fruits also reflects the healthy condition and the quality of the fruits. Thus, it is also significant to monitor the expansion of the plants. As shown in Figure 4e, Tang et al. developed a strain sensor based on carbon nanotube and graphite for plant growth monitoring [35]. The sensor was obtained by simply depositing graphite ink, and carbon nanotube (CNT) ink on a Latex substrate and solidifying under an ambient environment. With the synergistic reinforcement between CNT and graphite ink, this obtained strain sensor showed an increased sensing range compared to the pure graphite one. An all-in-one device composed of the sensor and a home-made readout circuit was used to make the real-time measurement of plant growth. The sensor was wrapped around a *Cucurbita pepo* to monitor the growth through its diameter. It showed evidence of a rhythmic growth pattern for the fruits: the fruits grow rapidly for seconds and then rest for seconds. Another work in Figure 4f also demonstrated the utilization of the strain sensor for plant pulse and growth monitoring through the diameter change [97]. This strain sensor was fabricated based on laser-induced graphene (LIG) and the design was inspired by the plants’ tendrils, which can adaptively wrap around the tomato stem without any paste or adhesive. This biomimetic tendril structure converts a direct stretching strain into the curvature effect and avoids strain deficiency from crack fracture. The expansion and shrink of the stem can generate resistance variation, which can be recorded by the strain sensor of which the signals were transmitted to smart phone wirelessly. The results show that the sensor system can accurately monitor the plant pulse to diagnose the growth and water state of tomato plants.

Overall, strain sensors play important roles in plant monitoring in the forms of adhesive-tapes assisted sensors, epidermal sensors, and wrapped sensors. They represent a significant advancement in the field of plant monitoring, offering real-time, accurate data on plant growth and stress responses. Their ability to detect minute deformations in plant tissues makes them invaluable tools for optimizing precision agriculture and improving crop-management strategies in agriculture.

## 5. Impedimetric Sensors for Plant Monitoring

Water content plays a crucial role in plant growth, as it is essential for photosynthesis, transpiration, and metabolic reactions [98]. It serves as the primary medium for physiological activities, playing a central role in maintaining cell structure, transporting nutrients, and regulating temperature in plants. Insufficient water can lead to slow plant growth, wilting leaves, reduced yields, and even death, while excessive water may also cause root hypoxia and disease [99,100,101]. Therefore, accurate and real-time monitoring of plant water content is crucial for agriculture and plant health management. There are various methods for monitoring water content, including optical reflectance, near-infrared spectroscopy [102,103], terahertz waves [104,105], and impedimetric sensors [106]. Among these, impedimetric sensors stand out for their simplicity and portability, offering real-time and long-term detection.

**Figure 5:**
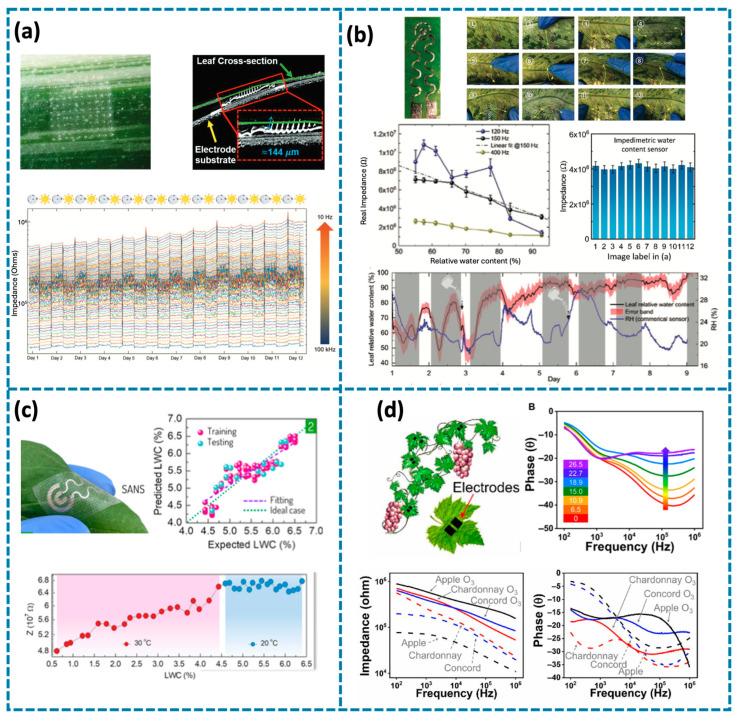
Impedimetric sensors for plant monitoring. (**a**) Microneedle array for monitoring impedance change of plants [107]. (**b**) Plant tattoo impedimetric water content sensor [108]. (**c**) Impedimetric sensor on plants for monitoring loss of water content [31]. (**d**) Vapor-deposited conducting polymer tattoos for identification of ozone damage in fruiting plants [17].

A microneedle-based (MN-based) bioimpedance sensor is proposed for monitoring the impedance change of plants due to lighting and hydration (Figure 5a) [107]. The material of the MNs is SU-8 2005 and polyimide PI-2611, which are biocompatible and capable of penetrating the cuticle of living plants without inducing any cytotoxic effects. The authors press the MNs on a freshly cut leaf of Arabidopsis thaliana and the optical image shows that the MNs can pierce the cuticle. As for the effects of MNs on plants, they mentioned that he puncture site becomes occluded and the microholes are sealed by the fourth day after wounding. This indicates that the MNs do not have any long-lasting detrimental effects on the integrity of the tissue. This MNs is used for water content monitoring. It was observed that impedance decreases as water content in the leaf increases. Over a 12-day MNs applied to Arabidopsis thaliana, a sudden increase in impedance was recorded during the transition from light to dark. This increase stabilizes at a level that remains higher than the values measured during the daytime photoperiod. The cyclical nature of the impedance fluctuations may be linked to the initiation of photosynthetic activity, which enables the leaf to function as a sink for water osmosis, subsequently lowering the impedance of the tissue. This MNs exhibit an average relative noise value of 3.83% in the open air, making them suitable for outdoor plant monitoring and applicable in farming.

The MNs mentioned above require penetration into the plant tissue for water content detection. Considering some methods that eliminate any harm to plants, there are also some non-invasive impedimetric sensors for plant water content monitoring. Yin et al. proposed a gold (Au)-polyethylene terephthalate (PET) tattoo impedimetric sensor for relative water content monitoring (Figure 5b) [108]. The sensor consists of a 1 mm-diameter circular pad electrode and a thin ring-like electrode to facilitate electrical impedance measurements, at 150 Hz, the impedance and the relative water content demonstrate an almost linear correlation. The impedance value shows minimal changes under various deformation conditions, demonstrating consistent performance. However, it tends to peel off after bending at a 90-degree angle more than 55 times. This tattoo sensor is attached to a leaf surface and the results can successfully show how leaf relative water content fluctuates in response to light and environmental conditions. Barbosa et al. designed the stand-alone Ni structure (SANS)-based electrodes for the detection of loss of water content (LWC), the SANS electrodes are attached to the leaf by using adhesive tape with high adhesion and long-term compatibility (Figure 5c) [31]. The electrodes are in serpentine traces with the ability to tolerate deformation. The SANS electrodes are attached on soy leaves for long-term LWC monitoring, under different degrees, 20 °C and 30 °C, the sensor shows different performance, under 20 °C the electrodes cannot sense the LWC. To address this problem, a machine learning method called supervised Sure Independence Screening and Sparsifying Operator, which combines prediction accuracy with computational speed and simplicity, was successfully applied to detect LWC at 20 °C with high accuracy. This approach offers a solution for mitigating temperature interference in LWC sensing and achieving more accurate LWC measurements.

Impedimetric sensors not only can be used for water content monitoring, but they can also be used for cellular ozone damage. Kim et al. fabricated Poly(3,4-dioxythiophene)-chloride (PEDOT-Cl) tattoos for long-term monitoring of cellular ozone damage in crops (Figure 5d) [17]. Ozone exposure uniquely alters the high-frequency (>10^4^ Hz) F5impedance and phase signals in leaves which is not observed with other abiotic stressors like drought. The PEDOT-Cl electrodes are deposited on the cutting grape leaves by oxidative chemical vapor deposition. When the leaf is exposed to varying levels of ozone, the impedance in the high-frequency range (10^4^ to 10^6^ Hz) increases linearly with ozone exposure, eventually reaching saturation at higher doses (>22.7 ppmh). The ozone-induced effects are even more pronounced in the phase-frequency plot, with a peak phase occurring at 10^5^ Hz. The electrodes are deposited on two different grape varieties and one common apple variety. The leaves of all investigated fruiting plants showed similar changes in impedance and phase signals at 105 Hz with ozone exposure. This method serves as living sensors for ground-level ozone, enabling timely interventions to reduce ozone damage and boost crop yield over multiple growing seasons.

## 6. Wearable Chemical Sensors for Plant Monitoring 

Plants respond to the surrounding environment and show their growth status by releasing plant metabolites, gas, and hormones. Metabolites like glucose and hormones like ethylene and jasmonic acid can directly reflect the metabolic activity, maturity, and stress response of plants [109,110,111,112,113]. By detecting these chemical signals in real-time, the dynamic changes of plant growth, such as photosynthesis efficiency, nutritional status, and growth rate of plants, can be revealed, thus providing real-time feedback for crop health management. The released VOCs can also serve as “early warning” signals of plant health, helping to monitor whether plants are suffering from diseases or environmental stress [114,115,116]. Another application for wearable plant sensors is to detect the residual concentration of pesticides on plant surfaces, thereby adjusting pesticide use at the appropriate time to reduce unnecessary impacts on plants [117]. In addition, harmful gases in the environment cannot be ignored, they can affect plant photosynthesis and respiration, leading to poor plant growth or even early death [118]. By detecting harmful gases in the environment, plant wearable sensors can provide information to help regulate the levels of greenhouse gases or pollutants and ensure a healthy growth environment for plants.

**Figure 6:**
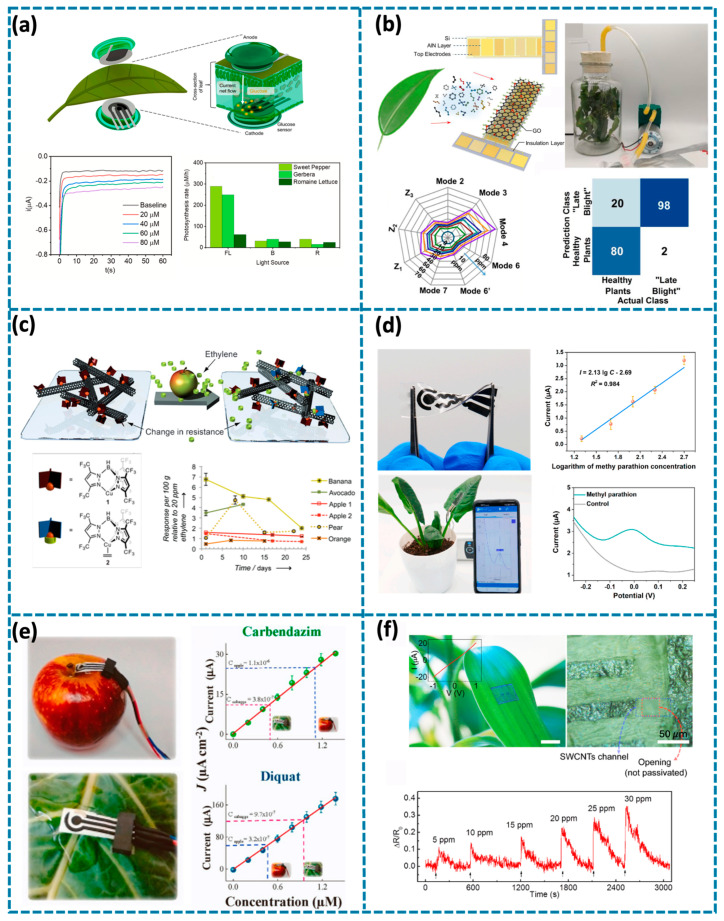
Wearable chemical sensors for plant monitoring. (**a**) Electrochemical biosensor for plant glucose sensing [45]. (**b**) Piezoelectric cantilever resonator for identification of VOCs from plants with disease [18]. (**c**) A reversible chemoresistive sensor for ethylene detection [38]. (**d**) Electrochemical biosensor for pesticide analysis [119]. (**e**) Non-enzymatic electrochemical sensors for the detection of different pesticides [120]. (**f**) The SWCNT–graphite sensor array on plants for monitoring DMMP in air [121].

Perdomo et al. recently fabricated a non-invasive wearable electrochemical enzymatic glucose-selective biosensor to monitor the glucose concentration of plants under different stresses; by applying the reverse iontophoresis method, glucose in plants can be extracted to reflect the associated physiological responses (Figure 6a) [45]. The glucose-selective sensor is based on a screen-printed three-electrode system which consists of the silver/silver chloride reference electrode, the Prussian blue carbon counter electrode, and working electrode. To realize glucose selective monitoring, glucose oxidase is coated on the working electrode. The total system is in a sandwich-type magnet with the glucose selective sensor, and the agarose-hydrogel-based negative terminal of the iontophoretic system serves as the cathode located at the bottom, then a polyvinyl alcohol hydrogel in-between the cathode and the leaf which is used to protect the leaf and transport the extracted glucose to the surface of the glucose selective sensor, on top of the leaf is the anode including another magnet with the agarose-hydrogel-based positive terminal. By applying a negligible current density (0.2 mA/cm^2^) between the anode and cathode, the sample of plant fluids from the leaves can finish triggering in 10 min. As the concentration of the glucose increases, the current output of the glucose-selective sensor increases. To verify the sensing performance, the authors put this system on three different plants (sweet pepper, gerbera, and romaine lettuce) to monitor their physiological responses associated with their glucose metabolism under low-light and low-high temperature stresses. The glucose concentrations of different plants are used to represent their photosynthetic rate. They characterize quantitatively and directly the behavior of this sugar on plants subjected to various stress conditions in real-time. 

In addition to glucose, the gas released by the plants generally consists of multiple VOCs and can also be a key biomarker for plant health monitoring. The mixtures of VOCs that healthy plants release should be different from the mixtures of VOCs that plants with diseases release; if we can identify them, then we can intervene to improve the plant’s health. Li et al. proposed a virtual sensor array based on an aluminum nitride piezoelectric cantilever with five groups of top electrodes to identify VOCs (Figure 6b) [18]. By integrating five electrodes, high amplitudes of multiple resonance peaks for the cantilever can be obtained. On top of the cantilever, graphene oxide (GO) is used as the sensing layer, since it can absorb the VOCs, and the mass effect can cause frequency shift in the cantilever. The frequency shifts of multiple resonant modes and changes of impedance value are used as the responses of the virtual sensor array to VOCs. These multidimensional responses create a distinct fingerprint for each VOC. This virtual sensor array is capable of distinguishing between healthy plants and those affected by late blight. As the concentration of (E)-2-hexenal, a key VOC biomarker for this plant disease, increases, both the resonant frequencies and impedance values decrease. With the help of machine learning, the accuracy of identifying plants with late blight and healthy plants is 87.5%, which shows the potential of plant disease monitoring.

Hormones like ethylene are a key signal for the developmental processes of plants. The concentration of ethylene can act as an indicator for harvesting fruits to avoid overripening and encourage plants to continue growing or flowering. Esser et al. developed a reversible chemoresistive sensor based on single-walled carbon nanotubes (SWNTs) to detect sub-ppm concentrations of ethylene (Figure 6c) [38]. To realize the selectivity of ethylene, a copper(I) complex 1 based upon a fluorinated tris(pyrazolyl) borate ligand is used to mix with SWNTs, it can interact with the surface of carbon nanotubes and influence their conductivity. When exposed to ethylene, 1 binds to ethylene and forms complex 2, and interaction with the SWNT surface decreases, which results in an increase in resistance of the SWNT network. The authors compared the ethylene emission from a selection of common fruits (banana, avocado, apple, pear, and orange) and found that there is an increased ethylene emission after the first week during the ripening of avocado and pear; after reaching peak ripeness, ethylene production decreased in banana, apple, and pear, with apples stored at room temperature senescing faster, while the non-climacteric orange showed consistently low ethylene emissions. This result is consistent with the respiration rate of climacteric fruit (banana, avocado, apple, and pear) and non-climacteric fruit (orange) during ripening. The system demonstrates strong potential for detecting the developmental processes of fruits.

In terms of ethylene detection, the ripening and aging process of fruits can be monitored, which can help to determine the best time to pick and ensure crop quality. Similarly, the detection of pesticide residues is also an important part of plant health management. By monitoring pesticide residues on the surface of crops, we can ensure healthy plant growth and reduce the harm of pesticides to consumers. Zhao et al. reported a flexible and stretchable plant-wearable biosensor to selectively capture and recognize the methyl parathion on crop surfaces (Figure 6d) [119]. LIG-based three-electrode is transferred to the PDMS substrate. To improve the sensing behavior, the gold nanoparticle layer is deposited on the working electrode, and to realize the selectivity of methyl parathion, organophosphorus hydrolase (OPH) equipped with biocompatible semisolid electrolyte is coated on the LIG-Au-OPH electrode surface. To see the performance of the sensor for monitoring the methyl parathion, the biosensor is attached to the spinach with the methyl parathion solution (100 μM) sprayed on the leaf of spinach. In contrast to the control experiments, clear peaks of p-nitrophenol are evident in the samples when exposed to methyl parathion, enabling qualitative detection of pesticide presence at specific times. This work provides a method for the acquisition of pesticide residue information; in addition, the pesticide residue information on the surface of real samples can be obtained through a smartphone equipped with the application Timely, which provides a portable system for real-time pesticide residue detection. Existing methods for detecting pesticide residues on plants are typically limited to qualitative analysis without providing accurate concentration data. Paschoalin et al. reported a three-electrode non-enzymatic sensor for detection of carbendazim and diquat with a detection limit of 43 and 57 nM, respectively, without interference from other pesticides (Figure 6e) [120]. The three-electrode system is fabricated by screen printing carbon inks on solution-blow spinning mats of poly (lactic acid) (PLA), which is flexible and low-cost. The oxidation peak of carbendazim appears at 0.1 to 0.8 V, while the oxidation peak of diquat appears at −1.1 to −0.4 V. Since the oxidation potentials of the two are far apart, they can be selectively detected in one experimental setting without interfering with each other. Differential Pulse Voltammetry (DPV) is suitable for carbendazim because its current signal is obvious in this range, while Square Wave Voltammetry (SWV) is suitable for diquat because its signal can be effectively detected within its specific potential range. After 100 μM carbendazim (or diquat) solution was sprayed on apples and cabbage and dried, electrochemical detection showed that the current signal of its oxidation peak changed with the concentration; by analyzing the DP and SW voltammograms, it was determined that the concentrations of carbendazim on apples and cabbage were 1.1 μM and 0.38 μM, and the concentrations of diquat were 0.32 μM and 0.97 μM, respectively. The effectiveness of the detection method was further verified with a control experiment using the standard addition method.

In addition to monitoring the plant metabolites and VOCs, some sensors are designed to detect harmful gases in the environment to further protect plant health. These sensors can perform real-time assessments to detect potential threats to plant health at an early stage. Lee et al. fabricated a single-walled carbon nanotube (SWCNT)–graphite sensor array to monitor dimethyl methylphosphonate (DMMP), a nerve agent simulant (Figure 6f) [121]. The sensor array consists of multiple blocks, each containing nine field-effect sensors. To enhance the sensitivity of DMMP detection, SWNT channels are functionalized with polypyrrole-(PPy-). The PPy-functionalized SWCNTs show reversible responses to DMMP with sensitivity even down to levels below the ppb range. As the concentration of DMMP increases, the resistance increases. The amine groups in DMMP transfer charge the p-type semiconductor SWCNTs, reducing the pore density in the SWCNTs, which leads to an increase in channel resistance. In addition, the DMMP molecules physically adsorbed on the SWCNTs also increase the contact resistance at the SWCNT–SWCNT joints. This SWCNT-graphite sensor array is transferred to a leaf surface of the ‘lucky bamboo’ by water solution to detect DMMP vapor in the air. The sensor array is flexible and can follow the topography of the leaf, which shows substantial promise for gas detection around the plants for plant health growth.

## 7. Multimodal Sensors for Plant Monitoring

During plant growth, leaf development, metabolite production, and volatile organic compound emissions are important indicators for assessing plant health and diagnosing potential diseases. In addition to monitoring the plant itself, their living environment significantly impacts healthy growth [122,123,124,125,126]. Therefore, it is critical to develop multimodal systems that can collect all the information simultaneously. Systems that integrate multimodal sensors can collect multidimensional data to provide a comprehensive understanding of the health of plants and their growth environment [127,128]. Sensors in a multimodal system may include strain sensors, VOC sensors, chemical sensors, and temperature sensors. When integrated for plant monitoring, these sensors enable the system to continuously track the plant’s diurnal growth cycle, detect hormones, secretions, and gases released during growth, and monitor key environmental parameters such as air quality and temperature. Such a multifunctional monitoring system can update the health status of plants in real-time and provide timely information, allowing human intervention such as rapid adjustment of temperature and air quality, to create optimal conditions for healthy plant growth, thereby increasing yield.

**Figure 7:**
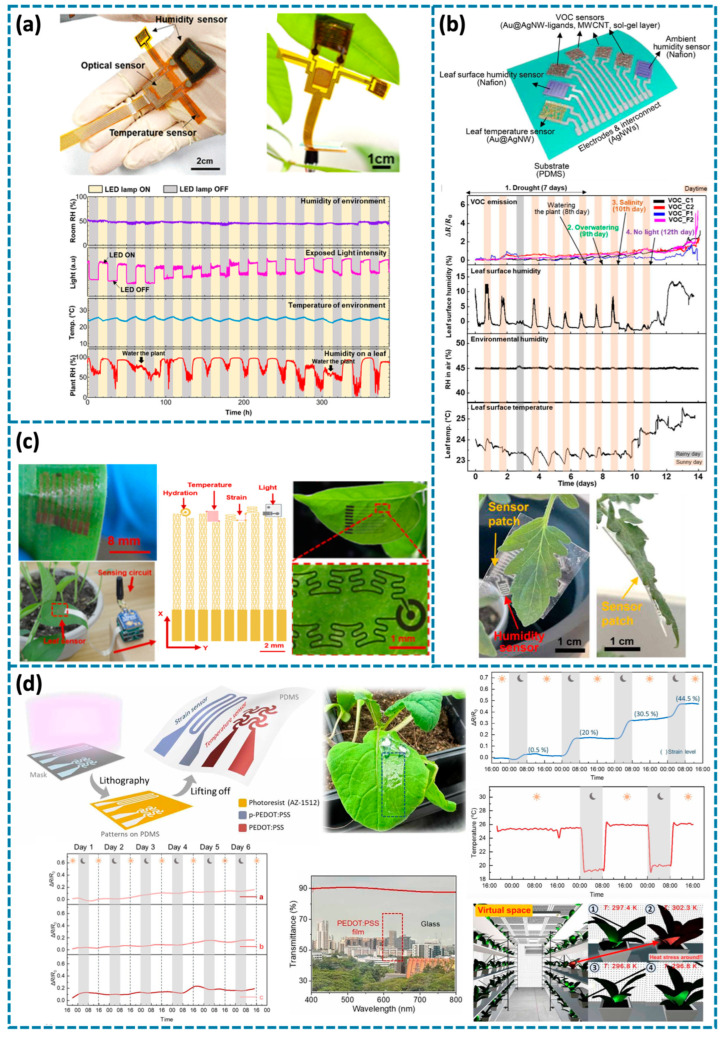
Wearable multimodal sensors for plant monitoring. (**a**) A multimodal flexible sensor system with humidity sensors, temperature sensors, and optical sensors for plant monitoring [39]. (**b**) A multimodal plant sensor patch with seven sensors for plant monitoring [40]. (**c**) A multifunctional sensor for monitoring plant growth and humidity, light illuminance, and temperature in the environment [33]. (**d**) An all-organic and transparent electronic skin for plant strain and temperature monitoring [32].

Lu et al. reported a multimodal flexible sensor system based on ZnIn_2_S_4_ (ZIS), which includes two humidity sensors for monitoring ambient humidity and stomata status, an optical sensor for detecting light irradiation intensity on plants, and a temperature sensor for environmental temperature (Figure 7a) [39]. The substrate of this device is the PI film, to avoid light shielding over the plant, the sensor system is attached to the lower epidermis, and the lower epidermis also has more stomas for testing. There is LIG surrounding the ZIS layer for water absorption, so the humidity sensor is not directly attached on leaves surface, which will not influence leaf growth. Temperature sensing is also important for plant monitoring. Since they use the same material for both the temperature sensor and the humidity sensor, to assess temperature effects on the humidity sensor, the device was tested at different temperatures with 30% constant humidity. The resistance remained stable across all temperatures. The sensor can be used for both humidity and temperature monitoring without interference. For the optical sensor, a transparent and flexible humidity-insulating film is applied to shield the sensor from ambient interference. The resistance decreases as light absorption increases, due to the generation of additional free electron–hole pairs in the ZIS film through optical absorption. The sensor system is attached to the leaf to investigate the plant growth behavior. Humidity, light, and temperature are three primary elements that influence plants’ transpiration. The plants are put under artificial light to simulate day and night in a constant temperature and constant humidity room. As the light density increases, the output from the humidity sensor shows that the plant humidity increases due to the stomates open for photosynthesis. When the lights are switched off, the stomata close to prevent water loss, leading to a decrease in humidity. The device can also detect that the relative humidity of the lower leaf is higher than that of the top leaf, which aligns with the natural laws of plant growth. This sensor system performs well in monitoring leaf humidity, as well as the light intensity and humidity of the surrounding environment.

During the process of plant growth, it is inevitable that plants will be attacked by various diseases. The development of sensors to detect these diseases plays a crucial role in ensuring timely diagnosis and treatment, which promotes healthy plant growth and ensures stable and efficient agricultural production. Lee et al. developed a multimodal wearable plant sensor patch that integrates seven sensors based on the PDMS substrate including four resistive VOC sensors, one capacitive leaf surface humidity sensor, one resistive leaf temperature sensor, and one capacitive environmental humidity sensor (Figure 7b) [40]. Gold-coated silver nanowires (Au@AgNWs) are deposited on the as-patterned interdigitated electrodes made of AgNWs for temperature sensor, Nafion are deposited for leaf surface humidity sensor and environmental humidity sensor, and functionalized Au@AgNW+ multiwalled carbon nanotubes (MWCNTs) are deposited for VOC sensors. The sensor patch is also attached on the lower epidermis of the leaf surface to avoid hindering stomata. It can be used for biotic stress and early pathogen detection. Fluorothiophenol, chlorothiophenol, bromothiophenol, and iodothiophenol are coated on the VOCs to selectively detect leafy aldehydes which are released by plants when they are damaged. These sensor patches are successfully attached on tomato plants for monitoring various abiotic stresses, including drought, overwatering, salinity, and darkness. In addition, the symptom of different pathogens, tomato spotted wilt virus (TSWV) and early blight on live tomato plants can be detected by the sensor patch. With the help of machine learning, the best sensor group can be found efficiently for disease detection and data show that VOC sensors play the most important role for detection, and then humidity and temperature sensors. This sensor patch provides a method for plant disease detection and plant monitoring.

Wearable sensors for plants should be as tiny, light, light-transmissive, and biocompatible as possible to avoid affecting the normal growth, photosynthesis, and respiration of plants. This design not only ensures a good combination of the device and the plant, but also effectively monitors the physiological state of the plant, thereby providing protection for its healthy growth. Zhao et al. designed a multifunctional sensor made of metal, CNT, and perforated silicone membrane for monitoring plant growth and humidity, light illuminance, and temperature in the environment (Figure 7c) [33]. The elements for various functions are interconnected by meshed structures, forming an island–bridge configuration. Each element features a hierarchical structure, allowing the entire device to grow synchronously with the leaf. This stretchable, lightweight, and biocompatible sensor permits light, gas, and water vapor to pass through, enabling direct attachment to the leaf surface for long-term monitoring, even in wet environments, with minimal interference to plant growth. Data collected from the sensor can be wirelessly transferred, providing a comprehensive tool for continuous environmental and physiological monitoring. Yang et al. presented a different multimodal electronic skin designed for monitoring plant strain and temperature, utilizing micropatterned poly(3,4-ethylenedioxythiophene): polystyrene (PEDOT: PSS) sulfonate on a PDMS substrate (Figure 7d) [32]. Notably, the sensor is transparent, all-organic, epidermal, and stretchable. The transparent sensor allows light transmission and gas while vapor-permeable material allows normal gas exchange, which are significant for photosynthesis and respiration to maintain normal plant growth. The e-skin is attached to the leaves of Epipremnum aureum; during the 65-day experiment, the plants remained healthy, and new roots and leaves emerged. The strain sensor can successfully detect diurnal growth patterns and varying growth rates under different abiotic stress and the temperature sensor can detect the environment temperature. In addition, the digital-twin plant monitoring interface is designed to enrich the visualization of the plant status.

## 8. Wireless Plant-Monitoring Systems

Based on previous discussions, current plant wearables, for their biocompatibility, peripheral supporting, sensitivity, and various functions, can now successfully implement this ability and carry out in situ detection in real conditions. Such sensors are always able to gather any physiological data and the growth or health status of a plant, thus providing relevant facts to the decision makers in agriculture. Advances in smart sensing technologies have done a great job in controlled indoor agriculture but the use of these technologies in outdoor agriculture is still at its developing stage [129,130]. This expansion is primarily driven by the need for cost-effective agricultural technology that can tap into the vast expanses of underutilized farmland, offering significant potential for enhancing yield, productivity, and sustainability in outdoor farming [131,132]. However, in large outdoor area settings, the traditional wired approach to reading signals from developed plant wearable sensors becomes a challenge [133,134]. Hence, attaching stand-alone sensors to signal processing and wireless communication systems is an important way in the development of smart outdoor farming technologies [135,136,137]. The analog signals of resistance, voltage, or frequency variations detected by plant wearable sensors can initially be converted into digital signals by a data-acquisition system. Subsequently, these signals are processed by an integrated microcontroller unit (MCU) module and wirelessly transmitted via a signal-transmission module.

**Figure 8:**
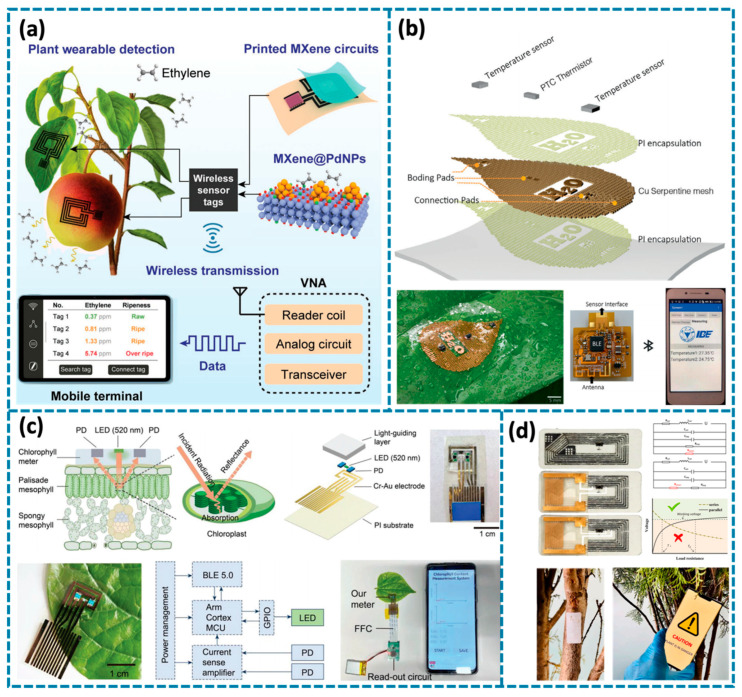
Wireless wearable sensors for plant applications. (**a**) Plant-wearable MXene-printed RF resonators for in situ ethylene detection [138]. (**b**) Thin, flexible electronic sensors with Bluetooth for real-time wireless sap flow monitoring in plants [46]. (**c**) Leaf-patchable, BLE-based wireless chlorophyll meter for non-destructive in situ monitoring [139]. (**d**) NFC-enabled wireless monitoring of α-pinene emissions in plants using a chemiresistor gas sensor [140].

At the moment, human body wearables incorporate wireless data-acquisition systems in order to perform their operations and most of the communication modes are near field communication (NFC), Bluetooth, and Wi-Fi. Plant wearable sensors adopt the same approach as well. In the study by Li et al., all-MXene-printed radio frequency (RF) resonators for wireless, plant-wearable, and in situ ethylene detection are reported (Figure 8a) [138]. They employ MXene, a two-dimensional conductive material that is also modified with palladium nanoparticles (PdNPs) to increase the detection of ethylene which is known to be an important plant-ripening hormone. With the addition of PdNPs, there is a change in the electrical resistance of the sensors which allows for the detection of the levels of ethylene by changes in the RF resonator frequency. These resonators are made by screen printing on a flexible form of the substrate which enables them to be directly placed on the surface of plants. The working mechanism relies on the fact that the introduction of ethylene molecules into the sensing area and binding of the molecules to the PdNPs alters the dielectric environment around the antenna, and this is expressed as a change in the resonant frequency of the antenna. The RF antenna is also used to send out these frequency shifts to a distance and data are coupled using RF protocols to ensure precision in transmission. This wireless transfer of these shifts is received and then assessed using a vector network analyzer which checks for frequency and amplitude changes with regard to ambient ethylene levels. The portfolio of the sensors shows high sensitivity and specificity, exhibiting a specific response of 1.16% to an ethylene concentration of 1 ppm and a detection limit of 0.084 ppm, characteristic of the sensors capable of detecting small changes in ethylene concentration. Furthermore, the sensors are calibrated within this resonant frequency range to maintain performance and reliability under different external factors. This system made it possible to monitor the amount of ethylene at the point of application in real time without modifying the physiology of the plant, making it possible to make progressive adjustments in the management of crops and the timings for harvesting. This and other similar scalable technologies offer great potential in improving data management in large agricultural fields without the cost and hassle associated with the use of wired systems. Chai et al. developed thin, flexible, wearable electronics for real-time sap flow monitoring in agricultural plants (Figure 8b) [46]. The configuration of the new sensor is based on an ultrathin, lightweight structure with serpentine copper conductors embedded into polyimide PDMS film. Such design enables biocompatibility and makes it possible for the device to integrate into plant surfaces readily without interference in physiological activities owing to being permeable to air, water, and light. The sensor dimensions are imperative to its operability as well as embedding into the plants systems, with about 20 μm in total thickness comprising a 10 μm PDMS layer which guarantees strong and reversible bonding which is beyond a soldering serpentine copper mesh about 6 μm in thickness all encapsulated with 2 μm thick polyimide. The process of assembly also considers the effect of the materials on the flexibility of the sensor and its effect on the physiology of the plant. The process employs a pair of aligned temperature sensors and a thermistor which positively responds to temperature variations and is placed between the two sensors. Upon activation, the thermistor is energized, and a temperature rise occurs at the thermistor, opening up the local environment. The temperature sensors are placed within the vascular bundles of the plant stem that Nepenthes exhibit. When the sap starts to flow through the system, the difference in temperature of the temperature sensors and that of the surrounding environment changes, where the sensor is adapted to calculate sap flow from differences in temperature in order to measure flows of between 5 and 415 µL. The data collected from the sensors are sent wirelessly using Bluetooth Low Energy (BLE) technology to a single integrated circuit that digitizes the information. The range that can be covered by the BLE module is often about 10 m, while the communication employs a frequency hopping spread spectrum to promote secure transmission of sensitive data. The smartphone connects with the sensor and the sensor can be set to time intervals when data are in two forms and are collected and displayed for the end user to analyze them in graphs and picture forms. This arrangement permits data collection for a number of sensors within agricultural phases in conjunction with foreseeing in real time the health of the plants as well as water utilization that is vital for better management of the farm along with research. Zhang et al. developed a leaf-patchable chlorophyll meter designed for non-destructive, in situ monitoring of chlorophyll content in plant leaves (Figure 8c) [139]. The fabricated device, weighing 0.2 g and with a thickness of 1.5 mm, is substantially less intrusive and lighter than conventional commercial chlorophyll meters, which typically require manual handling for measurements. The measurement of leaf light reflectance in plants is carried out using a monochromatic light-emitting diode (LED) and two photodetectors that are placed on a compliant substrate. The LED emits specific wavelengths that chlorophyll absorbs, while any unabsorbed light is reflected and detected by the photodetectors. The intensity of the reflected light, which inversely correlates with the chlorophyll content, allows the device to calculate a chlorophyll content index. This index shows a high coefficient of determination, indicating a strong linear relationship with actual chlorophyll levels. Equipped with BLE technology, the device transmits data wirelessly to a smartphone, enabling real-time monitoring and data logging. This lightweight, energy-efficient, and minimally invasive wireless sensor enhances the ability to detect plant stress early, allowing for more targeted agricultural interventions. Zheng et al. used NFC technology to enable wireless monitoring in agricultural settings with a chemiresistor gas sensor (Figure 8d) [140]. This sensor, designed to detect α-pinene emissions from Platycladus orientalis, includes a gold interdigital electrode and a composite film made of polyethylene-co-vinyl acetate and multi-walled carbon nanotubes. The sensor is highly selective and sensitive, with a minimum detection limit of 0.5 ppm. In response to 4 mm-wide bark damage, the sensor’s resistance increased by around 15%, and by 2% after 40 days of water stress. The NFC tag functions as a passive component in the system, altering its communication signal in response to changes in sensor resistance, which reflect varying α-pinene levels. These resistance changes, signaling plant stress, modulate the NFC signal, allowing real-time, non-invasive monitoring without needing wires or an external power source. In practical applications, the NFC tag allows the device to work effectively in the field, wirelessly transmitting data over short distances to NFC-enabled devices. This setup not only simplifies monitoring but also broadens the sensor’s use in remote or large-scale agricultural environments where traditional methods are less feasible.

## 9. Self-Sustainable Plant IoT Monitoring Systems

With the development of plant wearable devices, plant-sensing technologies have been widely applied in indoor and outdoor agriculture. To improve agricultural efficiency, the IoT was introduced to achieve remote monitoring and transmit data, allowing for timely human intervention to optimize plant growth conditions and enhance crop yield and quality [133,134,141,142]. However, there are some challenges to energy supply in outdoor environments, such as long-term use and difficult maintenance [135,136,143]. On the one hand, integrating self-powered sensors in the sensing system can help to lower the energy consumption; on the other hand, energy harvesters provide an environmentally friendly solution for powering the system. Energy harvesters face challenges like dependency on environmental conditions and the materials of the energy harvesters are harmful to the environment. For example, energy harvesters based on triboelectric nanogenerators and electromagnetics rely on vibration sources in the environment to drive, such as wind [144,145,146]. However, the vibration sources provided by the environment are often irregular, which can lead to instability in energy-harvesting performance [147,148]. In addition, energy harvesters such as piezoelectric energy harvesters contain lead zirconate titanate, which may cause environmental pollution in long-term use [149,150]. Therefore, it is crucial to develop an environmentally friendly energy harvester that can provide long-term stable energy for outdoor monitoring IoT systems.

**Figure 9:**
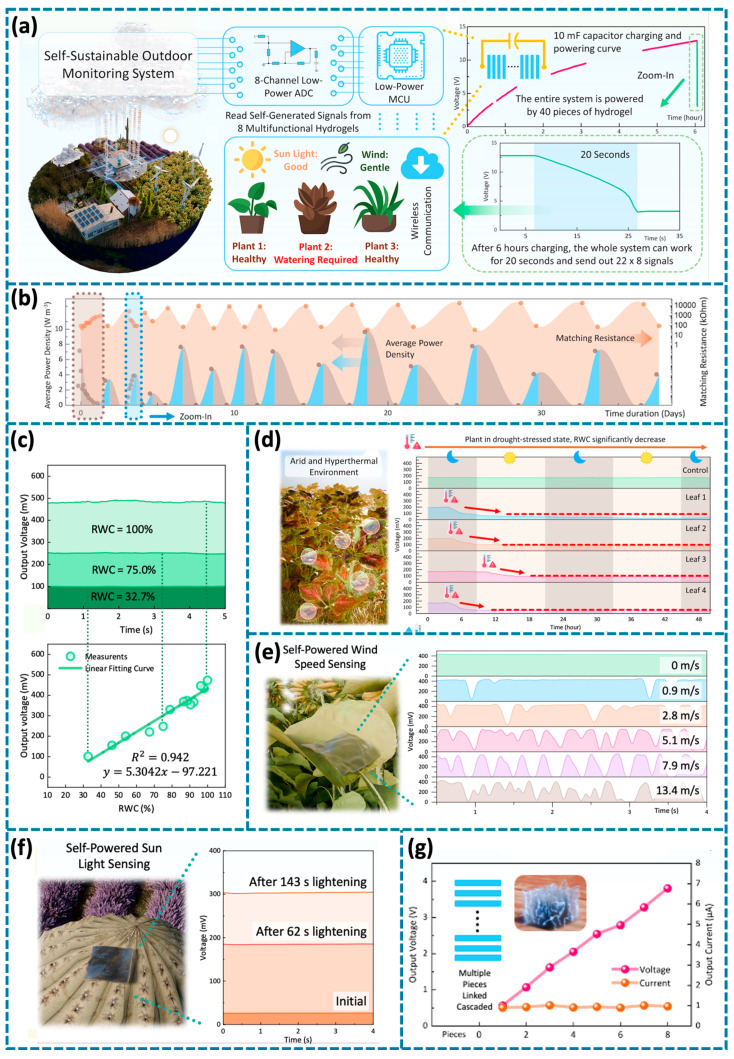
Self-sustainable plant IoT monitoring system [47]. (**a**) Overview of the multifunctional hydrogel-based self-sustainable IoT outdoor plant monitoring systems. (**b**) Durability and self-recovering of the hydrogel-based energy harvester under severe environment. (**c**) Multifunctional hydrogel for RWC monitoring. (**d**) Long-term IoT monitoring of RWC. (**e**) Multifunctional hydrogel for wind speed sensing. (**f**) Multifunctional hydrogel for sunlight sensing. (**g**) Cascading multiple pieces of multifunctional hydrogels to increase power output.

Guo et al. proposed a self-sustainable IoT outdoor plant monitoring system based on multifunctional hydrogel, as shown in Figure 9a [47]. The multifunctional hydrogel-based energy harvester consists of polyvinyl alcohol/polyacrylamide/lithium chloride/glycerol ionic hydrogel and copper–aluminum (Cu-Al) metal pairs as the electrode layers. Since the hydrogel is organic, low-cost, flexible, biodegradable, and biocompatible, it can be applied for non-invasive plant monitoring, making it safe for plants and environmentally friendly. With Cu-Al metal pairs, the redox potential difference allows ions like H^+^, Li^+^, and Cl^−^ to participate in reactions, generating a stable DC output. This enables the hydrogel to function as a self-powered sensor and an energy harvester. With the optimization thickness of 300 μm hydrogel, the hydrogel-based energy harvester can achieve an average power density of 1.9 W m^−3^. This hydrogel-based energy harvester can work with good durability and stability for 56.25 days in normal environments (24 °C, 60% RH), and the total energy density is 1.36 × 10^7^ J m^−3^. The authors also tested this hydrogel-based energy harvester under simulated severe outdoor environments (45 °C, 30% RH) for 37.67 days; due to water loss in the severe environment, there is an increase in matching resistance and a decrease in average power density. It is worth noting that this hydrogel-based energy harvester can recover to its normal state in a high-humidity environment (24 °C, 99% RH), since the hydrogel can automatically absorb the water content in the humidity environment, as shown in Figure 9b. The recovery ability of this hydrogel-based energy harvester allows it to operate long-term in outdoor environments with high durability and stability. To power the IoT system, 40 hydrogel pieces are cascaded to charge a 10 mF capacitor. As the number of cascaded hydrogel pieces increases, the output power also increases (Figure 9g). After 6 h of charging with 40 hydrogel pieces, the 10 mF capacitor reaches 13 V, which is sufficient to power a low-power MCU for approximately 20 s. This enables the wireless transmission of 8-channel data from the self-powered leaf relative water content (RWC) sensor, wind speed sensor, and sunlight sensor to the host. In addition, this multifunctional hydrogel can also be used as a non-invasive self-powered sensor for RWC monitoring (Figure 9c), wind speed sensing (Figure 9e), and sunlight sensing (Figure 9f). For RWC monitoring, the sensors can issue alerts when the plant’s RWC exceeds normal values (Figure 9d). The hydrogel-based sensor demonstrates long-term durability, operating reliably for 48 h in various environments, including normal conditions, arid and hyperthermal conditions, and rainy and humid conditions. This multifunctional hydrogel serves as a reliable, eco-friendly solution for sustainable IoT-based plant monitoring. Its dual function as an energy harvester and sensor allows for continuous, non-invasive monitoring in diverse environmental conditions. By powering low-energy devices to wirelessly transmit critical plant health data, this system provides a proactive approach to environmental monitoring and plant care, supporting timely interventions and promoting healthier growth. Due to the flexibility and biocompatibility of the hydrogel, the developed sensors and energy harvesters are environmentally friendly and safe for plants. This multifunctional hydrogel-based energy harvester and sensor provide a sustainable solution for continuous outdoor plant monitoring, addressing challenges of power supply and sensor instability under extreme conditions, providing more efficient, responsive, and eco-conscious agricultural practices. However, their multifunctional hydrogel-based sensor is limited to monitoring the relative water content (RWC) of plants and the microclimates. The plant monitoring system can expand its functionality to monitor plant status including monitoring leaf extension, detecting VOC emissions, stress response, etc. These enhancements would significantly increase the system’s versatility, enabling more comprehensive monitoring of plant health and environmental conditions.

## 10. Conclusions

With the growing global demand for food, intelligent plant-sensing technology has become a key tool to improve agricultural production efficiency. This article systematically introduces plant-sensing technologies based on different working principles, including piezoelectric ultrasonic sensors, optical-based sensors, wearable strain sensors, wearable impedimetric sensors, wearable chemical sensors, wireless plant monitoring systems, multimodal sensors, and self-sustainable plant IoT monitoring systems. These technologies can monitor plant physiological indicators, microclimate environment, and plant signaling molecules, greatly expanding the breadth and depth of plant health monitoring and making crop management more scientific and efficient. Piezoelectric ultrasonic sensors offer high sensitivity and non-invasive capabilities, but these sensors still face challenges such as outdoor robustness, environmental noise, and miniaturization for seamless integration with plants. To overcome these obstacles and sustain performance, it is necessary to improve material durability, implement advanced signal processing algorithms, and design more compact devices. Similarly, optical-based sensors are crucial for monitoring plant physiological states, but further innovation is needed to improve light penetration in dense foliage, boost durability against environmental stress, and enhance energy efficiency for sustainable, long-term applications. Future plant-sensing technologies, particularly wearable sensors, will develop in the direction of being non-invasive, highly biocompatible, flexible, miniaturized, highly sensitive, and light-transmitting. These wearable sensors will enable real-time monitoring of plant health and microclimates without disrupting natural plant growth, releasing toxins, or impacting photosynthesis and respiration. To achieve a comprehensive plant health assessment, future monitoring systems will develop in the direction of multimodal sensor systems, integrating multiple sensors such as temperature, humidity, gas, and strain sensors to monitor microclimates and the growth status of plant leaves and fruits, and detect hormones and VOCs produced by plants. This information from the multimodal sensing system can be used to prevent plant diseases and optimize harvest time and the plant growth environment, which will establish a robust foundation for precision agriculture and play a crucial role in enhancing global food security. With the development of outdoor agriculture, the need for autonomous energy supply is growing, making self-powered sensors and energy-harvesting systems an emerging trend. Self-powered sensors can minimize overall system power consumption and the stable, durable and self-sustainable systems that work normally in outdoor environments are also needed. Energy harvesters that operate independently of environmental conditions, such as hydrogel-based harvesters, represent a new trend by enabling continuous, long-term monitoring even in extreme weather conditions. The development of energy harvesters and self-powered sensors greatly helps the use of IoT systems outdoors. IoT will enable plant sensors to wirelessly transmit data to remote hosts in real time, reduce human costs, and help conduct efficient remote management and intelligent decision-making. With the upgrade of plant monitoring systems, rich data with complex information will be collected. The integration of artificial intelligence (AI) algorithms will enable deeper insights into plant growth patterns, early pest and disease detection, etc., significantly reducing the time and labor costs associated with manual analysis. AI will also enhance the accuracy of decision-making by analyzing large datasets, identifying subtle patterns, and generating predictive models for optimal growth conditions, resource allocation, and harvest timing. This intelligent approach will support precision agriculture, contributing to higher yields, more efficient resource use, and sustainable farming practices. In addition, digital twin technology will play a crucial role in enhancing plant-monitoring systems. Digital twin technology enables real-time mapping of sensor data to virtual models, creating virtual copies of physical systems. This allows for dynamic visualization and monitoring of plant growth processes and farm management. Through digital twin technology, farm managers can obtain detailed information about plant health and growth environments in real time at any time and place, ensure that crops grow under optimal conditions, and quickly respond to emergencies such as pests and diseases or extreme climate change, thereby achieving remote and efficient management. 

Overall, with the advancement of plant-sensing technologies, coupled with IoT, energy-harvesting technologies and AI algorithms, plant-monitoring systems will develop in a more autonomous, efficient, and sustainable direction. At the same time, the use of digital twin technology will allow dynamic, real-time visualization of plant growth processes, enabling farm managers to make data-driven decisions and proactively respond to emerging challenges. These advances will change agricultural practices and drive precision agriculture toward higher productivity, higher resource efficiency, and greater sustainability.
